# Increased maternal body mass index is associated with prolonged anaesthetic and surgical times for caesarean delivery but is partially offset by clinician seniority and established epidural analgesia

**DOI:** 10.1111/ajo.13277

**Published:** 2020-11-28

**Authors:** Sue Lawrence, Eva Malacova, David Reutens, David J. Sturgess

**Affiliations:** ^1^ The Centre for Advanced Imaging University of Queensland Brisbane Queensland Australia; ^2^ Mater Research Institute (MRI‐UQ) Mater Hospital Brisbane The University of Queensland Brisbane Queensland Australia; ^3^ QIMR Berghofer Medical Research Institute Brisbane Queensland Australia; ^4^ Mater Research Institute (MRI‐UQ) Princess Alexandra Hospital The University of Queensland Brisbane Queensland Australia

**Keywords:** anaesthesia, body mass index, caesarean section, neuraxial, obesity, obstetric

## Abstract

**Background:**

Obesity is associated with higher surgical and anaesthetic morbidity and difficulties.

**Aims:**

We aimed to investigate associations between maternal body mass index (BMI) and the in‐theatre time taken to produce an anaesthetised state or to perform surgery for caesarean delivery.

**Materials and Methods:**

Using the Strengthening the Reporting of Observational Studies in Epidemiology guidelines, we identified all women who underwent caesarean section at a single institution (2009–2015). The prospectively collected data arising from antenatal and peripartum care were analysed. Generalised linear regression was used to examine associations between maternal BMI and the time taken to anaesthetise the mother and the duration of surgery.

**Results:**

Of a total of 24 761 caesarean deliveries, 5607 (22.7%) women were obese at antenatal registration. In‐theatre anaesthetic preparation (18 vs 32 min, *P* < 0.001) and surgical duration (38 vs 52 min, *P* < 0.001) were longer in women with BMI ≥50 kg/m^2^ (BMI‐50) than those with normal BMI (BMI‐N). This difference remained significant after controlling for antenatal, intra‐operative and immediate postoperative variables. Modifiable variables were identified that may mitigate the effects of severe obesity. Senior obstetric and anaesthetic care were both independently associated with a significant reduction in mean in‐theatre anaesthetic preparation time and surgical duration, by 11 and three minutes respectively (*P* < 0.001), while epidural top‐up significantly lessened mean anaesthetic in‐theatre preparation duration by seven minutes (*P* < 0.001).

**Conclusions:**

Obese women had greater anaesthesia and surgery time, but the effect may potentially be mitigated by provision of care by experienced staff and prior establishment of epidural analgesia.

## Introduction

Obesity is increasingly prevalent (39% of all adults globally) and brings with it an increased burden of disease.^1^ Caesarean section under neuraxial anaesthesia is a relatively common, standardised operation that is performed on a population of patients with generally low co‐morbidity and for which there is routine prospective collection of associated data. It is therefore an ideal operation to study for the purpose of examining the relationship between severe morbid obesity and difficulties in generating neuraxial anaesthesia or performing intrabdominal surgery.

In 2017, 20% of Australian women who gave birth were obese at the start of antenatal care,^2^ with a body mass index (BMI) ≥50 kg/m^2^ occurring in 0.2% of all mothers.^3^ Studies identifying obesity as a contributor to difficulties in providing neuraxial anaesthesia in the birthing suite have generally grouped all obese (BMI ≥ 30 kg/m^2^) women together and compared them with normal BMI (18.5–24.9 kg/m^2^) cohorts.^4^, ^5^, ^6^ Obese women have also been shown to have higher rates of labour induction, failed induction of labour, deliver by caesarean section, have longer surgical durations, and be at increased risk of maternal and neonatal complications.^2^, ^14^ Results of an adequately powered investigation of a bariatric subset with regard to outcomes of caesarean delivery is yet to be added to our knowledge base.^3^, ^4^, ^5^, ^15^, ^16^, ^17^, ^18^

This study uses routinely collected, institutional data pertaining to all women delivering by caesarean section and their infants to determine if a woman’s BMI is an independent variable influencing the time taken to generate anaesthesia for caesarean section and to perform the surgery.

## Materials and Methods

The Mater Mothers’ Hospital and Mater Mothers’ Private Hospital are co‐located facilities within the Mater Health, South Brisbane campus. This is a maternity service with the highest capability ranking.^19^ In 2017, 3% of all Australian neonates were delivered on this campus.^2^, ^20^ Our maternity service delivery is streamed into two co‐located care models; publicly funded, multidisciplinary health care and private/health insurance funded, specialist obstetrician‐led care.

After gaining Mater Misericordiea Ltd Human Research Ethics Committee and Mater Governance Office site specific approvals (HREC/17/MHS/93 and RG‐17‐085) with waiver of consent, institutional maternal, obstetric, operating theatre, neonatal and admission databases were queried for data relating to all gestations ending in caesarean section with live‐born infants at Mater Health Service, South Brisbane from 1 January 2009 to 31 December 2015.

### Antenatal variables

Collected antenatal data included maternal height, pre‐pregnancy weight, age at delivery, residential postcode, smoking status, illicit substance use, healthcare model, plurality of pregnancy and gestational age at booking. Experienced senior anaesthetic and obstetric clinicians were always the primary care providers for caesarean section of women in the private healthcare model, whereas in the multidisciplinary care model there was a mix of senior and lesser experienced clinicians. Socio‐economic status was derived from maternal residential postcode,^21^ then divided into quintiles for analysis.

Calculated BMI was classified according to American Heart Association recommendations specific for the surgical setting.^22^ This system builds upon the World Health Organization BMI classification framework^23^ by further subdivision of ‘Class III obese’ (BMI ≥ 40.00 kg/m^2^) into three separate categories, creating Class III obese (BMI = 40.00–49.99 kg/m^2^), Class IV obese (BMI = 50.00–59.99 kg/m^2^) and Class V obese (BMI ≥ 60.00 kg/m^2^).

### Peripartum variables

Gestational age at delivery was collected. Duration of antenatal care was calculated by subtracting booking from delivery gestational age. Mothers receiving up to one week of tertiary antenatal care were deemed to have received intrapartum care only at the study site.

Anaesthetic categories were limited to spinal, epidural top‐up, combined spinal epidural or general anaesthesia as there were no cases of de novo epidural only anaesthetics for caesarean section in this investigation. Epidural top‐up was assigned if medication was administered through a pre‐existing epidural catheter sited for labour analgesia, regardless of whether the medications were administered before transfer to or in the theatre suite. If labour epidural top‐up was attempted but converted to general anaesthesia, it was classified as a general anaesthetic. Any combination of any epidural anaesthesia and spinal anaesthesia was classified as a combined spinal epidural (CSE) anaesthetic.

Several classifications of operative delivery were collected. Degree of urgency was recorded as a dichotomous elective/emergency variable and using the Royal Australian and New Zealand College of Obstetricians and Gynaecologists promulgated 1–4 categorisation.^24^ Caesarean section as a surgical procedure is classified according to the site of uterine incision and denoted as lower segment or classical.^25^

In‐theatre anaesthetic preparation duration was deemed to be from the anaesthetic start time to the surgical start time. Anaesthetic start time was noted as soon as the patient had both entered the theatre suite and had an anaesthetist in attendance. Surgical duration began at procedure start time and ended at procedure end time. Procedure start time marks both the end of anaesthetic preparation and the beginning of surgical duration. Procedure end time was noted at application of the wound dressing or alternatively, initiation of a subsequent surgical procedure. Post‐caesarean in‐theatre duration, total in‐theatre anaesthetic care duration and duration of post‐anaesthesia care unit care was also analysed. Anaesthetic start time was classified dichotomously into sociable or unsociable hours, where unsociable hours were daily from 23:00 to 7:00 hours.^26^

Immediate maternal postoperative intensive care unit (ICU) admission, length of maternal inpatient admission as compared to industry funding allocation were collected to indicate maternal postoperative course. Suspected fetal problem and neonatal Apgar score at five minutes were captured; as well, neonatal nursery admission from theatre was recorded as metrics of infant well‐being.

### Statistical analysis

Generalised linear models with the identity link function were used to assess the association of BMI with the duration of in‐theatre anaesthesia preparation and of caesarean section procedure both in univariate and multivariable analyses. All variables with origin in the antenatal period were included in the modelling. Complete‐case analysis was used for instances of missing data. A two‐tailed *P*‐value of < 0.05 was considered statistically significant. SAS version 9.4 (SAS Institute Inc., Cary, NC, USA) was used for linear modelling. R version 3.6.1 with RStudio version 1.2.5019 was used for Pearson’s χ^2^ test with and without Yates’ continuity correction, Fisher’s exact test and Welch two‐sample *t*‐testing.

## Results

Of the 24 980 caesarean sections with live‐born infants in the total dataset, 219 (0.9%) were removed due to lack of data on maternal height or weight, resulting in a final dataset of 24 761 records. At the time of booking, 22.7% of women (5607) were classified as obese (BMI ≥ 30 kg/m^2^) and 108 (0.4%) had a BMI ≥ 50 kg/m^2^.

Demographic, antenatal and perinatal characteristics are summarised in Table 1. For demographic comparison of means against the BMI 18.5–24.9 kg/m^2^ category (BMI‐N), we combined obesity classes IV and V into BMI ≥ 50 kg/m^2^ (BMI‐50).

**Table 1:** Demographic, antenatal and perinatal characteristics by body mass index classification

Body mass index classification (kg/m^2^)	Total	Underweight (<18.5)	Normal and overweight (18.5–29.9)	Obese Classes I, II (30–39.9)	Obese Class III (40–49.9)	Obese Class IV (50–59.9)	Obese Class V (≥60)
Antenatal							
Total†	24 761 (100)	380 (1.5)	18 774 (75.8)	4784 (19.3)	715 (2.9)	92 (3.7)	16 (0.06)
Age (years)‡	32.8 [14–59]	30.8 [15–45]	32.9 [14–57]	32.8 [16–59]	32.7 [20–47]	33.0 [20–47]	32.7 [21–40]
Socio‐economic status‡	3.0 [1–5]	2.8 [1–5]	3.1 [1–5]	2.7 [1 ‐ 5]	2.3 [1–5]	2.1 [1–5]	1.6 [1–4]
Current smoker†	1204 (8.9)	22 (12.2)	763 (7.5)	341 (12.8)	65 (17.0)	9 (16.4)	4 (36.4)
Illicit substance use†	1827 (7.4)	35 (9.4)	1337 (7.2)	362 (7.6)	85 (12.1)	7 (7.9)	1 (6.3)
Multidisciplinary healthcare model†	10 610 (42.8)	223 (58.7)	7560 (40.3)	2295 (48.0)	459 (64.2)	61 (66.3)	12 (75.0)
Singleton pregnancies†	23 443 (94.9)	362 (95.2)	17 796 (94.8)	4520 (94.5)	665 (93.0)	84 (91.3)	16 (100)
Gestational age at booking (weeks)‡	20.0 [2–41]	17.7 [6–41]	19.9 [3–41]	20.5 [3–41]	20.3 [2–39]	22.0 [6–39]	27 [17–36]
Antenatal care duration (weeks)‡	17.9 [0–39]	19.6 [0–34]	18.0 [0–37]	17.2 [0–39]	17.1 [0–36]	15.5 [0–32]	7.8 [0–22]
Intrapartum care only (<1 week)†	501 (2.1)	9 (2.4)	328 (1.7)	130 (2.7)	27 (3.8)	6 (6.5)	1 (6.3)
Antenatal care duration if >1 week (weeks)‡	18.2 [1–39]	20.1 [1–34]	18.2 [1–39]	17.7 [1–39]	17.8 [1–36]	16.4 [1–32]	8.3 [1–22]
Gestational age at birth (weeks)‡	37.9 [23–42]	37.3 [24–42]	38.0 [23–42]	37.7 [23–42]	37.4 [32–42]	37.4 [27–41]	34.8 [27–39]
Very preterm birth (<32 weeks)†	987 (4.0)	33 (8.7)	684 (3.6)	217 (4.5)	45 (6.3)	5 (5.4)	3 (18.8)
Preterm birth (<37 weeks)†	3715 (15.0)	85 (22.3)	2 647 (14.0)	805 (16.8)	146 (20.4)	22 (23.9)	10 (62.5)
Time durations (min)							
In‐theatre anaesthetic preparation duration‡	18.6 [0–121]	17.6 [0–75]	18.0 [0–108]	19.9 [0–121]	24.9 [1–115]	30.6 [2–81]	42.3 [14–106]
Surgical duration‡	39.5 [9–379]	39.2 [11–92]	38.5 [9–379]	41.6 [9–224]	48.6 [19–216]	50.9 [23–105]	57.8 [29–102]
Post‐caesarean in‐theatre duration‡	8.2 [0–311]	8.4 [0–111]	8.0 [0–311]	8.7 [0–277]	9.4 [0–185]	12.9 [0–177]	20.75 [4–104]
Post‐anaesthesia care unit duration‡	56.0 [4–586]	59.0 [12–292]	55.5 [4–586]	56.6 [8–465]	60.2 [9–266]	74.3 [31–478]	73.4 [40–105]
Total in‐theatre anaesthetic care duration‡	66.4 [20–457]	65.2 [29–153]	67.2 [24–457]	72.8 [31–359]	82.9 [37–344]	94.3 [40–260]	120.9 [58–192]
Peripartum							
Anaesthetic							
Spinal†	14 933 (60.3)	234 (61.6)	11 498 (61.2)	2819 (58.9)	344 (48.1)	33(35.9)	5 (62.5)
Combined spinal epidural†	4032 (16.3)	50 (13.2)	2901 (15.5)	840 (17.6)	199 (27.8)	32 (34.8)	10 (31.3)
Epidural top‐up†	4676 (18.9)	69 (18.2)	3575 (19.0)	897 (18.8)	118 (16.5)	17 (18.5)	0 (0)
General†	1120 (4.5)	27 (7.1)	800 (4.3)	228 (4.7)	54 (7.6)	10 (10.9)	1 (6.3)
Type of caesarean section and postoperative							
Emergency†	11 907 (48.1)	230 (60.5)	9006 (48.0)	2275 (47.6)	344 (48.1)	43 (46.7)	9 (56.3)
Category 1†	2670 (11.1)	67 (18.2)	2064 (11.3)	468 (10.0)	58 (8.4)	9 (10)	4 (25.0)
Classical†	291 (1.2)	7 (1.8)	197 (1.0)	67 (1.4)	16 (2.24)	1 (1.1)	3 (18.8)
Unsociable hours†	3105 (12.5)	65 (17.1)	2,434 (13.0)	521 (10.9)	76 (10.6)	7 (7.6)	2 (12.5)
Maternal intensive care unit admission†	225 (0.9)	3 (0.8)	144 (0.8)	56 (1.2)	15 (2.1)	4 (4.3)	3 (18.8)
Prolonged maternal length of stay†	3732 (15.2)	48 (12.7)	2744 (14.8)	772 (16.2)	138 (19.5)	25 (27.2)	5 (31.3)
Fetal problem†	9393 (37.9)	150 (39.5)	6830 (36.4)	2010 (42.0)	346 (48.4)	46 (50)	11 (68.8)
Apgar < 7 at 5 min†	638 (2.6)	8 (2.1)	448 (2.4)	144 (3.0)	29 (4.1)	9 (9.8)	0
Nursery admission†	4747 (19.2)	98 (25.8)	3,356 (17.9)	1069 (22.3)	190 (26.6)	27 (29.3)	7 (43.8)

^†^ Number (%).

^‡^ Mean [range].

### Demographic characteristics

The mean maternal age at delivery, singleton pregnancy rate and use of illicit substances did not differ across the BMI range. BMI‐50 women were significantly more likely to be from a lower socio‐economic quintile (*P* < 0.001) and utilise publicly funded multidisciplinary health care (68% vs 41%, *P* < 0.001) as compared with BMI‐N women.

### Antenatal characteristics

The mean duration of antenatal care was shorter for BMI‐50 women when compared to BMI‐N women (14 vs 19 weeks, *P* < 0.001). They tended to book later (23 vs 19 weeks, *P* < 0.001) and were more likely to have their care transferred to the study hospital for intrapartum care (7% vs 2%, *P* < 0.001). BMI‐50 women were significantly more likely to have a preterm delivery (30% vs 14%, *P* < 0.001) but the difference to BMI‐N women in relation to very preterm delivery was not statistically significant (7% vs 4%, *P* = 0.074). The overall effect of booking and delivery differences was a significantly shorter duration of antenatal care (14 vs 19 weeks, *P* < 0.001) and this persisted after removal of the women transferred solely for intrapartum management (15 vs 19 weeks, *P* < 0.001).

### Peripartum characteristics

It was more complicated to anaesthetise BMI‐50 women: there was no difference from BMI‐N with regards to epidural top‐up but they were more likely to have a general anaesthetic (10% vs 5%; *P* = 0.015) or a CSE (39% vs 15%; *P* < 0.001) with a commensurate decrease in spinal anaesthesia rates (35% vs 61%; *P* = 0.004).

Surgical characteristics were similar between BMI‐50 and BMI‐N. There was no significant difference with reference to urgency classifications, surgical procedure or delivery during unsociable hours. Postoperatively BMI‐50 differed from BMI‐N in that a greater percentage were transferred from theatre to ICU (7% vs 1%, *P* < 0.001) and were also more likely to remain as an inpatient beyond the standardised length of stay (28% vs 15%, *P* < 0.001).

Health outcomes of babies from the BMI‐50 subgroup were less assured. The fetuses of BMI‐50 mothers as compared to those of the BMI‐N were more likely to be allocated an alert regarding a potential problem (53% vs 36%, *P* = 0.026), register an Apgar score of less than seven at five minutes (2.4% vs 8.3%, *P* = 0.008) and be admitted to a nursery (32% vs 18%, *P* = 0.005).

**Figure 1:**
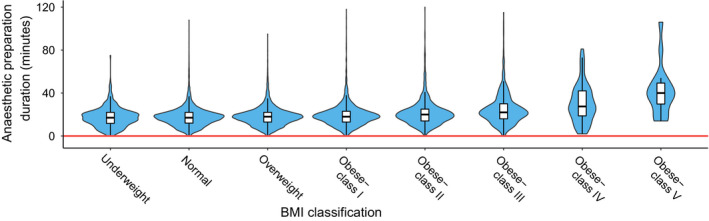
Violin plot of in‐theatre anaesthetic preparation duration by maternal body mass index (BMI). Violin plot shape illustrates probability density (wider = higher probability). White bars with black lines delineate median and interquartile range. Reference red line is at zero minutes. Note the change in shape with maternal BMI ≥ 50 kg/m^2^(Obese – Classes IV and V) as well as rising median values.

**Figure 2:**
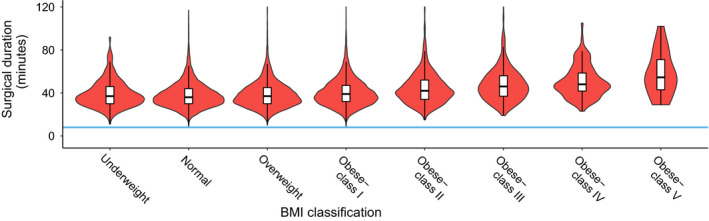
Violin plot of surgical duration by maternal body mass index (BMI). Violin plot shape illustrates probability density (wider = higher probability). White bars with black lines delineate median and interquartile range. Reference blue line is at eight minutes. Note the rising first interquartile and median values with increasing maternal BMI.

More time was used in the theatre suite at every stage of a caesarean section for the BMI‐50 subgroup. Greater obesity classification as compared to BMI‐N was significantly associated with longer in‐theatre anaesthetic preparation duration (Table 2 and Fig. 1). The mean in‐theatre anaesthetic preparation duration for BMI‐50 women was 32 min as compared with BMI‐N women at 18 min (*P* < 0.001). The increase in in‐theatre anaesthetic preparation duration was modest initially as maternal BMI increased but escalated as obesity rose beyond 40 kg/m^2^. The probability distribution for anaesthetic preparation also altered as obesity became more severe indicating increased variation of anaesthetic efficiency in the obese Class IV and V BMI groups (Figs 1 and 2).

**Table 2:** Associations of body mass index with in‐theatre anaesthetic preparation duration and surgical duration for caesarean section before and after adjustment

	In‐theatre anaesthetic preparation duration (min)				Surgical duration (min)			
	Univariate	*P*‐value	Multivariate	p‐value	Univariate	*P*‐value	Multivariate	*P*‐value
	β (SE)		β (SE)		β (SE)		β (SE)	
Intercept	17.8 (0.1)		21.0 (0.2)		38.3 (0.1)		44.4 (0.3)	
Body mass index (kg/m^2^)								
Underweight (<18.5)	−0.2 (0.5)	<0.001	−0.4 (0.4)	<0.001	0.9 (0.7)	<0.001	−0.5 (0.6)	<0.001
Normal weight (18.5–24.9)	Reference		Reference		Reference		Reference	
Overweight (25–29.9)	0.4 (0.1)		0.2 (0.1)		0.5 (0.2)		0.6 (0.2)	
Obese Class I (30–34.9)	1.5 (0.2)		1.1 (0.2)		2.3 (0.3)		1.6 (0.2)	
Obese Class II (35–39.9)	3.4 (0.3)		2.3 (0.3)		6.2 (0.4)		4.6 (0.4)	
Obese Class III (40–49.9)	7.1 (0.4)		5.4 (0.3)		10.3 (0.5)		7.6 (0.5)	
Obese Class IV (50–59.9)	12.8 (1.0)		10.9 (0.9)		12.6 (1.4)		9.4 (1.3)	
Obese Class V (≥60)	24.5 (2.4)		19.0 (2.1)		19.5 (3.4)		12.6 (3.0)	
Anaesthetic used for surgery								
Spinal			Reference	<0.001			Reference	<0.001
Epidural top‐up			−6.7 (0.2)				0.2 (0.3)	
Combined spinal and epidural			3.1 (0.2)				−0.4 (0.2)	
General anaesthetic			−2.9 (0.3)				1.5 (0.4)	
Type of caesarean section								
Elective lower segment			Reference	<0.001			Reference	<0.001
Emergency lower segment			5.6 (5.9)				−12.8 (8.6)	
Elective classical			8.7 (1.1)				20.2 (1.6)	
Emergency classical			7.8 (6.0)				−6.1 (8.6)	
Classification of urgency of caesarean section								
Category 1 (most urgent)			−11.7 (5.9)	<0.001			10.8 (8.6)	<0.001
Category 2			−6.9 (5.9)				13.0 (8.6)	
Category 3			−4.9 (5.9)				12.9 (8.6)	
Category 4 (elective)			Reference				Reference	
Unknown			−5.2 (5.9)				12.3 (8.6)	
Multidisciplinary or specialist obstetrician‐led healthcare model								
Specialist obstetrician‐led care (private health care)			−2.9 (0.1)	<0.001			−10.5 (0.2)	<0.001
Hospital medical/midwifery care (public health care)			Reference				Reference	
Immediate postoperative ward								
Post‐anaesthesia care unit			Reference	<0.001			Reference	<0.001
Intensive care unit			7.6 (0.6)				11.7 (0.8)	
Anaesthesia initiated during unsociable hours: (23:00–7:00 hours)								
No			Reference	0.001			Reference	<0.001
Yes			−0.6 (0.2)				1.5 (0.3)	
Gestational age at birth (weeks)								
<28 (extremely preterm)			−0.2 (0.6)	<0.001			0.7 (0.9)	0.035
28–31 (very preterm)			0.5 (0.4)				−0.9 (0.5)	
32–36 (moderately preterm)			1.2 (0.2)				0.5 (0.3)	
37–40 (term)			Reference				Reference	
≥41 (post‐term)			−0.4 (0.2)				−0.3(0.3)	
Socio‐economic status (quintiles)								
<20th percentile (most disadvantaged)			0.4 (0.2)	0.060			0.5 (0.3)	0.001
20–39th percentile			0.1 (0.2)				0.7 (0.2)	
40–59th percentile			0.04 (0.2)				0.5 (0.2)	
60–79th percentile			0.3 (0.2)				0.04 (0.2)	
≥80th percentile (most advantaged)			Reference				Reference	
Maternal age (years)								
<20			0.05 (0.5)	0.337			−2.6 (0.7)	<0.001
20–29			Reference				Reference	
30–39			0.1 (0.1)				1.3 (0.2)	
≥40			−0.3 (0.2)				2.5 (0.3)	
Smoking status								
Non‐smoker			Reference	0.6			Reference	<0.001
Ex‐smoker			−0.1 (0.2)				−0.2 (0.3)	
Smoker			0.2 (0.3)				−0.4 (0.4)	
Unknown			0.1 (0.1)				−3.1 (0.2)	
Tertiary antenatal care duration (weeks)								
≥1			Reference	0.834			Reference	0.003
<1			0.1 (0.4)				−1.5 (0.6)	
Unknown			−0.7 (1.4)				4.8 (2.1)	
Illicit substance use								
No			Reference	0.075			Reference	0.470
Yes			0.4 (0.2)				−0.4 (0.3)	
Unknown			−1.1 (0.7)				0.2 (1.1)	
Multiple pregnancy								
No			Reference	0.076			Reference	0.715
Yes			0.5 (0.3)				−0.1 (0.4)	

Similarly, greater obesity classification as compared to BMI‐N was associated with a longer time taken to perform a caesarean section (Table 2 and Fig. 2). The mean surgical duration was 38 min at the nadir for the BMI‐N group and rose to 52 min (*P* < 0.001) for BMI‐50.

The mean in‐theatre post‐caesarean section duration was significantly longer for BMI‐50 as compared with BMI‐N women (17 vs 11 min, *P* = 0.002). Total in‐theatre anaesthetic care was also prolonged (101 vs 67 min, *P* < 0.001). Time spent in the post‐anaesthesia care unit also followed this trend (74 vs 56 min, *P* = 0.001).

The association between in‐theatre anaesthetic preparation duration or surgical duration and BMI remained significant even after controlling for all collected temporal covariates (Table 2). Multivariate linear regression analysis identified factors that were associated with shorter in‐theatre anaesthetic preparation duration: care delivered by more experienced clinicians, obstetrician (11 min, *P* < 0.001) or anaesthetist (3 min, *P* < 0.001) and choice of anaesthesia. On average top‐up of a labour analgesic epidural was seven minutes quicker (*P* < 0.001) and general anaesthesia four minutes (*P* < 0.001) than the reference spinal anaesthetic in achieving adequate anaesthesia.

## Discussion

This investigation illustrates the effect of severe obesity upon theatre suite time utilisation. Increasing BMI was associated with increased in‐theatre anaesthetic preparation and surgical duration. The effect on in‐theatre anaesthetic preparation duration was more pronounced as the BMI increased beyond 40 kg/m^2^. Our study period ran concurrent with three comparable Australian investigations: the Mum SIZE study (*n* = 1475),^15^ Australasian Maternity Outcomes Surveillance System (AMOSS) super‐obesity cohort study (*n* = 991)^3^ and a Brisbane pilot study by Eley et al. (*n* = 63).^4^ Our results confirm and build upon aspects of each of these studies.

When compared to Mum SIZE,^15^ our study adds detail and expands observations by inclusion of more antenatal variables and non‐elective caesarean sections, separation of the in‐theatre epochs, and consideration of more detailed subdivision of BMI.^22^ Our results confirm their findings of longer anaesthetic and surgical times required for caesarean delivery with increasing obesity, but offers additional understanding regarding the non‐linearity of change as BMI rises beyond 40 kg/m^2^ and that there are important factors that may be pertinent to the efficient care of severely morbidly obese women.

The AMOSS super‐obesity cohort study^3^ was designed to determine prevalence and characteristics relevant to pregnancy outcomes of women with a BMI > 50 kg/m^2^ or weight > 140 kg. Their calculated prevalence for this level of obesity was 0.2%; our equivalent prevalence was 0.4%. The observed difference was both due to the transfer of high BMI women to a specialist obstetric facility in our study and to ours being a caesarean delivery‐only cohort. Our results confirm their findings with regard to maternal age, socio‐economic disadvantage, smoking status, plurality of pregnancy, model of health care, birth before 37 weeks gestation, emergency caesarean section, general anaesthesia and ICU admission rates.

The pilot study from a nearby institution in Brisbane analysed a cohort of women with a BMI of ≥40 kg/m^2^.^27^ Our results confirm their findings with regard to rates of category one urgency caesarean section and surgical duration. In contrast to our results, they reported a significant difference with regard to the ability to convert labour epidural analgesia into epidural anaesthesia.

Our study also confirms the findings of a study investigating the time taken to access the neuraxis (*n* = 427). The authors reported that increasing BMI strongly predicted more difficulty in palpation of bony landmarks and less flexion of the lumbar spine.^16^ This may, in part explain the increased in‐theatre anaesthetic preparation time observed in higher BMI groups of our study.

We gathered no information on prior non‐obstetric or obstetric surgery, and it may have confounded the observed association of increased surgical duration with increased maternal age.^28^ Other limitations include the poor recording rate of smoking status for women in the specialist obstetrician healthcare stream as noted by previous authors^29^ and the absence of urgency classification^24^ data in some cases at the beginning of the collection period.

Our investigation was a large, single centre, cross‐sectional study of all caesarean sections performed at a major obstetric and neonatal health facility over a period of seven years. The resulting large and comprehensive dataset enabled detailed examination of the effect of maternal BMI categories on the time required to perform two specific phases of caesarean sections.

In summary, BMI was positively correlated with both the time it takes to generate anaesthesia for caesarean section and the time required to perform the surgery. The effect of BMI on in‐theatre anaesthetic preparation duration was more pronounced as the BMI increased beyond 40 kg/m^2^. This association was independent of maternal age, socio‐economic or smoking status, illicit substance use, healthcare model, pregnancy plurality, gestation at booking or delivery, duration of antenatal care, type of anaesthetic or caesarean section, surgery at unsociable hours or maternal state requiring transfer to ICU. Experienced anaesthetic and obstetric clinicians may lessen the effect of increasing severity of obesity upon both anaesthetic preparation and surgical duration. The presence of labour epidural analgesia that was successfully converted to epidural anaesthesia also lessened the effect of BMI upon in‐theatre anaesthetic preparation duration; this reinforces current guidelines for the intrapartum management of obese women.

## Funding

This research did not receive any specific grant from funding agencies in the public, commercial, or not‐for‐profit sectors.

## Availability of data and materials

The data, ‘The influence of maternal body mass index on anaesthetic and surgical times for caesarean delivery’, that support the findings of this study are available from UQ eSpace (https://doi.org/10.14264/uql.2019.507) but restrictions apply to the availability of these data. Data are available upon reasonable request.
